# Integration and Exploitation of Sensor Data in Smart Cities through Event-Driven Applications

**DOI:** 10.3390/s19061372

**Published:** 2019-03-19

**Authors:** Manuel Garcia Alvarez, Javier Morales, Menno-Jan Kraak

**Affiliations:** Geo-information Processing Department, ITC Faculty, University of Twente, Hengelosestraat 99, 7514 AE Enschede, The Netherlands; j.morales@utwente.nl (J.M.); m.j.kraak@utwente.nl (M.-J.K.)

**Keywords:** event-driven applications, IoT applications, reference architecture, geographic events, complex event processing, sensors, smart cities

## Abstract

Smart cities are urban environments where Internet of Things (IoT) devices provide a continuous source of data about urban phenomena such as traffic and air pollution. The exploitation of the spatial properties of data enables situation and context awareness. However, the integration and analysis of data from IoT sensing devices remain a crucial challenge for the development of IoT applications in smart cities. Existing approaches provide no or limited ability to perform spatial data analysis, even when spatial information plays a significant role in decision making across many disciplines. This work proposes a generic approach to enabling spatiotemporal capabilities in information services for smart cities. We adopted a multidisciplinary approach to achieving data integration and real-time processing, and developed a reference architecture for the development of event-driven applications. This type of applications seamlessly integrates IoT sensing devices, complex event processing, and spatiotemporal analytics through a processing workflow for the detection of geographic events. Through the implementation and testing of a system prototype, built upon an existing sensor network, we demonstrated the feasibility, performance, and scalability of event-driven applications to achieve real-time processing capabilities and detect geographic events.

## 1. Introduction

Smart cities are urban environments where the use of IoT devices spans across many disciplines such as environmental monitoring, traffic management, and early-warning systems [1,2,3,4]. Sensors are the backbone of a smart city because they enable situational and spatial awareness [5,6,7] for real-time monitoring of dynamic geographic phenomena. Some examples are traffic, the movement of people, air pollution, and energy consumption [8,9]. Such a wide range of applications is possible because the exploitation of data collected by sensors serves as a proxy to understand spatiotemporal patterns [10], and it also enables the generation of information to support decision making [5,6].

The exploitation of data relies on the implementation of information services. However, existing research in smart cities gives more attention to the technological challenges of providing software components for information services [11,12,13], and they rarely give attention to the provision of relevant information for improving productivity and efficiency in the use of resources, or the citizen’s quality of life [14,15]. Some works address the implementation of mechanisms to collect and access data from heterogeneous sources [16], while other works develop tools that implement efficient data processing [17,18] or develop algorithms for specific data analytic routines [19,20]. Geographic information is a key element in the exploitation of big data in smart cites because it provides contextual information for a myriad of applications [21]. Nevertheless, existing information systems and services offer software components with no or limited ability for spatial data analysis [16,22], even when spatial information plays a crucial role in decision making for many disciplines in the domain of smart cities [9,23].

To address the limitations listed above, we propose a generic approach for enabling spatiotemporal capabilities in information services for smart cities and a reference architecture for the design of “event-driven applications”. Event-driven applications are information services that use geographic events [24,25,26] as abstractions to extract relevant spatiotemporal information from sensors networks, and monitor dynamic geographic phenomena. Geographic events provide contextual information to event-driven applications by encapsulating spatiotemporal properties of a dynamic geographic phenomenon. Geographic events are detected by implementing complex event processing systems [27,28], a technology that provides big data analytics and real-time processing.

Some examples of geographic events are a car accident, a peak in the concentration of air pollutants, and a drop in the consumption of electricity. However, the use of such technology poses a challenge when defining data abstractions and implementing the geographic components that are required to represent and detect geographic events using sensor networks. Through the definition of a reference architecture, we provide an abstract framework to design applications that require the integration of data from heterogeneous sources, efficient data management for thousands of sensors, continuous data processing, and big data analytics [29,30,31].

Our approach presents several advantages compared with the state-of-the-art. First, the approach focuses on providing services that remove the burden and complexities of extracting information from heterogeneous sensors. Second, our reference architecture enables the development of information services with spatial functionality, data interoperability, scalability, and real-time processing. Third, event-driven applications seamlessly integrate IoT technologies, complex event processing, and spatiotemporal analytics. Lastly, in comparison with works in the field of geographic information [32,33], our event-driven processing approach provides a generic workflow for the detection of geographic events at different levels of abstraction.

We begin this paper by summarizing relevant research around three topics: big data in smart cities, event processing, and geographic events (Section 2). In Section 3, we describe key aspects of the design of event-driven applications and present the processing workflow. Section 4 describes RASCA, a reference architecture for the development of event-driven applications. In Section 5, we describe the prototype of a system that complies with RASCA and detects geographic events for the case of air pollution monitoring. In Section 6, we present the results of a series of performance test of our prototype. We discuss the results and limitations of our approach in Section 7, and present our main conclusions and future research directions in Section 8.

## 2. Related Work

The development of IoT solutions for smart cities demands a multidisciplinary perspective, and it often requires the integration of multiple technologies [34]. Two of the core steps in the development of IoT applications are the integration of IoT devices and their data, and the analysis of such data for a targeted service [35]. Recently, the continuous generation of data by IoT sensing devices has increased the need for information services that provide data integration, interoperability, high-performance data processing, scalability, and real-time analytics [29]. In this section, we highlight some the efforts to provide capabilities for the integration and exploitation of data from sensors in smart cities, and we describe some of the state-of-the-art for the detection of geographic events in the domains of event processing and geographic information.

### 2.1. Big Data in Smart Cities

Smart cities have been a topic of research for at least ten years and yet there is no agreement on the models that cities should implement to become smart. Smart city models usually provide definitions, dimensions, and strategies to achieve smartness. Some models depict smart cities as an approach to promote sustainable economic growth and high quality of life through the development of human and social capital using the communication infrastructure [14]. A broadly accepted model suggests that smart cities should focus on the development and use of technologies in six application domains: economy, mobility, environment, human capital, governance, and livability [36,37]. Another model suggests that, besides the development of technologies to solve urban problems, smart cities should also include instruments to monitor and evaluate urban phenomena [38]. Even though there is no universal agreement on the definitions and dimensions of a smart city, most models coincide that smart cities have the following characteristics [39]:

- IT infrastructure and high-tech for promoting efficiency, productivity, and quality of life.
- Social inclusion, especially when it comes to governance.
- Sustainability and efficiency in the use of resources are main drives.

Sensors enable pervasive sensing [7] and continuous data collection of dynamic geographic phenomena, and sensors are an essential part of the IT infrastructure in smart cities. Examples of dynamic geographic phenomena are traffic, air pollution, movement of people, and weather [10,40]. Nevertheless, it is widely recognized that the pervasive use of sensors in cities contributes to the big data problem. For example, the SmartSantander sensor network collects 3000 observations per day, in the city of Santander in Spain [2,41]. Another case is the sensor network in Barcelona, which already collects 1.3 million observations per day, and plans to deploy sensors within 100 km^2^ of the city, estimating a data collection rate of approximately 722 million observations per day [29].

A recent trend in data collection is the adoption of humans as sensors through dedicated platforms or social media [20]. Aguilera et al. [16] developed a platform that promotes the use of human sensors for implementing participatory data services, while Sacco et al. [42] described an information service for mining Tweets with the intention of understanding human behavior. The influence of social media platforms and the ubiquity of smartphones contribute to the challenge of exploiting heterogeneous data sources.

Software platforms and reference architectures provide developers with frameworks and tools for designing and implementing applications in smart cities. Santana et al. [43] reviewed the architectural characteristics of some of the initial smart city platforms, and they identified real-time processing, scalability, context awareness, interoperability, and security as requirements and challenges in the development of such platforms. Recently, Shahat Osman, A. [44] reviewed the architectural characteristics of frameworks for big data analytics in smart cities, and identified similar key requirements.

Other existing works, related to the exploitation of data from sensors, consider important one or more of the following architectural characteristics [45,46,47]:

- the architecture pattern;
- the computing environment, centralized or decentralized;
- the type of scalability, vertical or horizontal;
- the type of processing, batch or real-time;
- the type of analytics, historical or real-time; and
- the inclusion of security and privacy features.

Table 1 presents a comparison between a selection of works regarding reference platforms and architectures for smart cities.

Significant progress has been made regarding the interoperability between sensor networks. The Sensor Web Enablement (SWE) standards promote the design and implementation of sensor interfaces and services, which make data discoverable and accessible for cross-platform applications [48]. One of the earlier standards, the Sensor Observation Service (SOS), describes a web service to store and retrieve observations [49]. More recently, the SensorThings API standard describes an Application Programming Interface (API), which applies the paradigm of IoT to sensing devices. The SensorThings API is expected to substitute the SOS as it implements more efficient communication protocols [50].

When processing and analyzing large amounts of data, performance and scalability are two common requirements for information services. A trending approach is the use of cloud computing to scale and balance the load of collecting data using hundreds of thousands of sensors [51]. Another approach is to integrate big data technology to achieve higher performance in data analytics [52]. A more recent approach suggests the use of fog computing, an architectural approach in which part of the data processing happens closer to devices and the end-users, as a complementary technology to deal with managing and processing large amounts of data in decentralized environments [53]. Performance has also been addressed through the design of system architectures that aim to control the behavior of a systems to maximize performance and manage the use of computational resources. Some examples are the data analytic framework described by Puiu et al. [54], and the semantic reasoning approach described by D’Anello et al. [18].

An approach to improve the response time of a system is the implementation of real-time processing frameworks such as stream processing [17]. Event processing is a type of stream processing that supports the integration of multiple data sources, and implements pattern matching for the detection of high-level information abstractions, so-called events [27]. Nevertheless, the detection of complex patterns usually presents implementation challenges. To overcome some of those challenges, Moraru et al. [55] combined event processing and machine learning to detect complex patterns in data streams generated by IoT devices. The following section provides more details about the novelties and current challenges in event processing.

### 2.2. Complex Event Processing

Event processing is a mature technology for the detection of events. Event processing consists of a set of operations to detect, transform and combine different types of events [56,57]. Events are usually typified as simple or composite events. A *simple event* is an event that is not the result of combining other events. A *composite event* results from specifying relations between simple or other composite events [58]. Complex Event Processing (CEP) describes a set of operations used to define and detect composite events [59].

CEP is also an active field of research when it comes to the processing of data streams [58]. Cugola et al. [28] considered CEP as a part of the core functionality of systems that exploit data from multiple and distributed sources, and that require continuous processing of data streams. According to Windley [60], CEP enables the development of reactive web applications, and it leverages the ideas behind the Semantic Web. Through this approach, software entities and devices act as event emitters and consumers, while dedicated web services provide event processing capabilities. Web services provide continuous data processing by implementing a dynamic data-static query approach, in this approach streams of data are contentiously evaluated against a set of queries, and only data that satisfy those queries are processed further.

In traditional CEP, events are detected by applying algorithms that match time (*temporal matching*) and other attributes (*attribute matching*) of a data stream. Complex events are the result of the aggregation of simple events based on time windows [27,57]. However, the detection of geographic events also requires being able to match data streams based on the geographic location (*spatial matching*) of the sensor or device that generate them. To the best of our knowledge, there has only been one attempt to explicitly apply CEP to the detection of geographic events [32]; however, the authors only presented a proof of concept that implements spatial overlay for spatial matching.

Table 2 presents a comparison of the functionality implemented by current CEP engines. These engines were selected based on their open source philosophy and their claim as general-purpose CEP engines. Most CEP engines implement a heterogeneous data model and provide functionality for temporal and attribute matching. CEP engines that adopt a heterogeneous data model are more flexible and allow the representation of events with a variable number of properties, i.e., the number of tuples or records representing an event does not have to be the same for all events inside the engine. In contrast, CEP engines that implement a homogeneous data model impose strict data formats for representing events, therefore reducing data interoperability [28,61].

Regarding spatial matching, CEP engines provide only partial spatial functionality. T-Rex implements an “area of interest” predicate to constrain the detection of an event to a geographic region [62], and the Siddhi CEP only provides functionality for performing geocoding [63]. When it comes to performance, T-Rex, Siddhi CEP, and Apache Flink report the highest processing rates (events/second).

Performance metrics represent the efficiency of a CEP engine to detect events [58]. Performance metrics include *throughput*, i.e., the number of input data units processes per unit of time; *time-cost*, i.e., the time required to process one unit of input data; and *detection latency*, i.e., the time between the occurrence of the event in the physical world and its detection by the CEP engine [71]. Another type of latency is *communication latency*, which refers to the delays generated by transferring detection messages through a communication network. Communication latency is especially relevant when CEP is implemented as a distributed system or as part of real-time systems. Current efforts to improve the efficiency of CEP engines focus on query planning and runtime execution [72,73].

There have not been enough efforts to implement CEP technologies as part of information services in smart cities. Moreover, it is only recently that such technology has attracted attention in the geographic information domain [15,74]. However, its relevance in the development of smart city applications has been pointed out in a variety of use cases, for example in systems that manage the logistics for transporting goods [75] or that coordinate the communication between IoT devices and sensors [33,76,77]. Other examples include the automated management of smart building [78,79] and the management of smart grids [80]. CEP is becoming increasingly popular for supporting real-time processing in applications for smart cities.

### 2.3. Detection of Geographic Events

The detection of geographic events has been approached using a variety of techniques, for instance through the development of algorithms that detect temporal and spatial relations in time series [81]. Other techniques use pattern matching or similarity indexes to detect changes in the temporal and spatial properties of geographic phenomena [26,82]. Moreover, visual analysis and interactive tools are used to support data analysts to detect geographic events manually [83,84]. Despite the suitability of such techniques for detecting geographic events, they present limitations regarding their feasibility to be automated or to provide the robustness and performance required by real-time processing.

The processes of detecting events using CEP requires unambiguous definitions for types of events to be detected. Nevertheless, the definition of a *geographic event* varies between experts in geographic information. According to Peuquet et al. [26], a geographic event is a representation of “a change in state (i.e., change in property, attribute, or value) that can be denoted as such for some feature or location or set of features or locations”. However, according Gatalsky et al. [83] and Worboys et al. [25], a geographic event is a world entity represented by a discrete spatial object with a relatively short duration (e.g., construction of a road, urban expansion, and a person’s life). In contrast, event processing experts prefer more general definitions. Luckham [56] defined an event as “anything that happens or is contemplated as happening", and Cugola et al. [61] as things of interest that occur instantaneously at some points in time.

Therefore, in the context of this work, we define a geographic event as an information object representing the occurrence of specific values that define a change of state in the properties of an observable dynamic geographic phenomenon, within a particular space-time window. A geographic event has the following characteristics:

- It can be represented as a spatial object, i.e., an information object containing spatial information.
- It has a finite but variable duration.
- It represents a change in properties or values related to a geographic phenomenon.
- An event is declared as of interest by a particular user or for a specific application.

Section 3.4 presents a formal representation for this definition of a geographic event.

## 3. Designing Event-Driven Applications

The purpose of addressing the design aspects of smart city applications is to provide a generic approach to the development of applications that support real-time decision making in smart cities. By generic, we mean that the approach is not limited to applications in a specific domain, e.g., traffic management, environmental monitoring, etc. Rather, we describe an approach that can be applied to a range of application domains and cases. The approach relies on sensors as the main sources of data, while CEP is adopted as a suitable technology to achieve real-time processing and to detect geographic events.

The generic approach in question should comply with the following requirements.

1. Propose a formal definition for event-driven applications and their most general purpose.
2. Identify the users, who define which types of information are required for decision making and decide on the specific purpose of an event-driven application.
3. Develop a generic data processing workflow for the detection of geographic events.
4. Formalize data representations for modeling the flows of information in the data processing workflow.
5. Develop a reference architecture that describes the core functionality required by event-driven applications.

In the remainder of this section, we describe our solutions to comply with Requirements 1–4. Requirement 5 is addressed in Section 4.

### 3.1. Event-Driven Applications

Event-driven applications detect the occurrence of relevant geographic events to support decision making. The general purpose of an event-driven application is to provide users with information of geographic events and support real-time decision making. Real-time decision making means that users take decisions about the most relevant problem at hand considering the most recent information available. For example, consider a *pollution alert application* that uses the sensors deployed in an urban environment to collect data about air quality, detect high concentrations of pollutants, and immediately alert users when the levels surpass certain safety limits. Informing users about those geographic events prompt them to take timely decisions to protect their health. Event-driven applications have the following characteristics:

- They rely on sensors as a continuous source of spatial and temporal data to monitor dynamic geographic phenomena in an urban environment.
- They enable the detection of geographic events by implementing temporal, spatial and attribute matching.
- They provide users with information services in real-time.

Within event-driven applications, we classify sensors as: in-situ, mobile, and human sensors. In-situ sensors have a fixed location; this includes sensors usually attached to some of the cities structures (e.g., lamp post and buildings). Mobile sensors change location, these sensors are usually attached to vehicles (e.g., cars, buses, UAVs, etc.). Human sensors are smartphone users who provide data about incidents happening in the city (e.g., vandalism to public property), through social media or dedicated data platforms.

### 3.2. Users

Users play an essential role in the design of any application. In event-driven applications, users’ interests and motivations define which information—geographic phenomena and events—is relevant for supporting decision making. Users adopt one of three roles: city administrator, businessman, and citizen.

For a city administrator, the motivations behind the need of information focus on monitoring the current status of a city as a whole, anticipating or responding to problematic situations, and on planning future developments. Some examples include members of the local government, rescue teams, or urban planners. For a businessman, the motivation lies in identifying business opportunities and developing business strategies, for example identifying niche markets, planning a marketing campaign, or implementing new business models. Lastly, for a citizen, the motivations are typically transient and respond to the needs and desires of the individual, for example planning how to spend leisure time or reduce commute times, or just asking about the weather forecast. Table 3 summarize the user’s roles and their motivations.

### 3.3. Event-Driven Processing Workflow

The users’ information requirements decide the purpose of an event-driven application, and the decision of what geographic events and phenomena are relevant. A processing workflow describes a set of steps required to transform data into information. Event-driven applications require processing steps to retrieve data from sensors deployed in an urban environment, perform event detection, communicate the occurrence of events, and provide information about events to the users.

**Figure 1:**
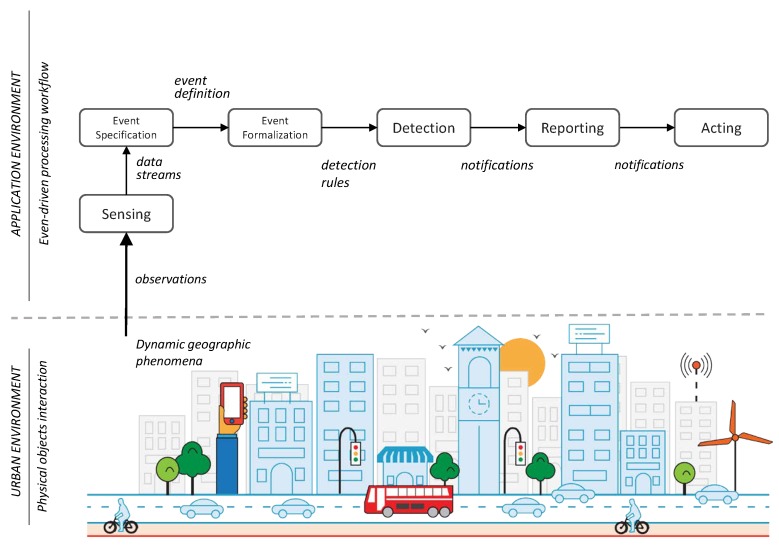
The event-driven processing workflow: a generic data processing workflow for event-driven applications.

Figure 1 depicts the event-driven processing workflow: a generic data processing workflow for event-driven applications. Data flows from the urban environment where a geographic event happens, to the application environment where event detection takes place. The *sensing* step collects observations of dynamic geographic phenomena and packs them as data streams. Observations and data streams are data representations of the values measured by sensors; data representation are explained in Section 3.4.

The event specification step declares the properties and constraints of a geographic event using a formal specification mechanism, i.e., a well-defined set of properties and values. The event formalization step focuses on transforming an event specification into detection rules, i.e., patterns that data streams must be matched to identify the occurrence of a geographic event. The detection step applies CEP to data streams. CEP evaluates detection rules against each data streams and it creates notifications containing relevant information about the occurrence of a geographic event. The reporting step communicates notifications to event consumers, which are software entities capable of interpreting notifications.

Lastly, in the acting step, notifications are consumed by event consumers. Event consumers are programmed to use event notifications based on an application’s specific purpose, for example display event metadata, trigger data analysis routines, or create other types of events.

### 3.4. Data Representations

Data representations model information in the event-driven processing workflow. Observations and data streams depict data collected by sensors, and event definitions and detection rules describe the properties and constraints of a geographic event, while notifications embed information about the occurrence of events. These data representations are formally defined as follows.

**Observations**: An observation is as set *o* of name-value pairs representing data collected by a sensor, such that

$$o=\{name_{1}:value_{1},name_{2}:value_{2},\ldots name_{n}:value_{n}\}$$

An observation contains, at least, an identifier for the sensor, a measurement, the sensor’s location, a time, and metadata describing the phenomenon being observed.

**Data streams**: A data stream *d* is represented as a sequences of one or more observations belonging to a sensor *s*, such that

$$d_{s_{1}}=\left(o_{1},o_{2},o_{3},\ldots o_{n}\right)$$

**Event definitions**: An event definition is a formal representation of a relevant geographic event describing a set of attributive, spatial, and temporal characteristics [15]. The users’ information requirements determine the relevance of a geographic event. Each characteristic is represented as a set of name–value pairs describing an event’s properties. The event’s attributive properties, $D_{p}$, include a name, a list of phenomena of interest, and a conditional statement to be met by the phenomena. The event’s spatial properties, $D_{s}$, include constraints of the geographic space in which the event is relevant, i.e., extent and spatial granularity. The event’s temporal properties, $D_{t}$, include constraints of the time within which an event is relevant, i.e., time window and duration.

Thus, each set of properties is represented as,

$$D_{p}=\left\{name:value,phenomena:\left[value_{1},value_{2},\ldots value_{n}\right],condition:value\right\}$$

$$D_{s}=\left\{extent:value,granularity:value\right\}$$

$$D_{t}=\left\{time-window:value,duration:value\right\}$$

Consequently, the definition of a geographic event *e* is represented as the union set of all its properties with a global event identifier (id), as following,

(1) $$e=\{id,\left(D_{p}\cup D_{s}\cup D_{t}\right)\}$$

**Figure 2:**
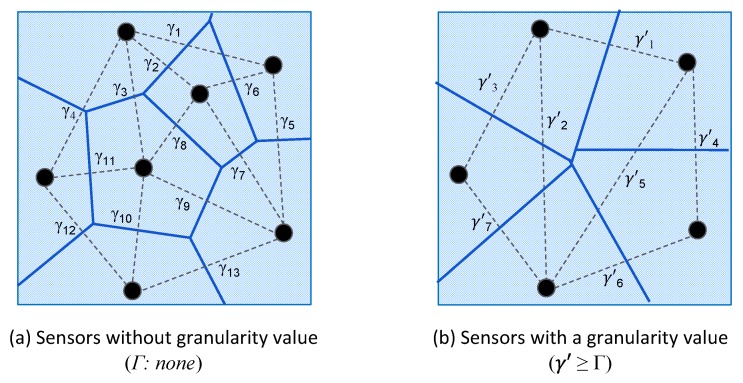
Examples of the effect of granularity values in the definition of event: (**a**) sensors when no granularity value is defined; and (**b**) effect of defining a granularity value on the selection of sensor.

Spatial properties are represented by an extent and a granularity. The *extent* specifies an area in geographic space within which the happening of an event is relevant. Values for an extent are represented using a geometry and a distance. A geometry can be of the type *LineString*, *MultiString*, *Polygon*, or *MultiPolygon* [85]. A distance is used to define a buffer zone around the exterior of a geometry, and is optional for the types *Polygon* and *MultiPolygon*. The *granularity* property specifies the level of detail in geographic space required for an event. If we assume that the observations provided by a set of sensors are valid for the space immediately close to the location of the sensors, then the partition of the geographic space into regions using a Voronoi diagram represents the granularity for an specific set of sensor. Figure 2 illustrates this.

In event definitions, the granularity property provides a mechanism to control the size of the regions around the location of sensors which has a direct effect on the selection of sensors for a specific event. The value for the granularity property is defined by a required minimum distance (Γ) between the locations of a set of sensors, such that $\Gamma >MIN(\gamma_{1},\gamma_{2},\ldots \gamma_{n})$, where γ represents the initial distances between sensors in a set of sensors. Figure 2a shows an example when no granularity is defined for a given set of sensors, and Figure 2b shows the effect of defining a granularity value in the selection of sensors and the partitioning of the geographic space.

The temporal properties of an event are represented using a *time-window* and a *duration*. A time-window tw can be defined in three ways: as a continuous time interval using start and end times, such that tw=[start,end]; as a cyclic time using a time interval and a number of repetitions, such that tw={[start,end],repetitions}; and as a time pattern using a collection of non-overlapping time intervals, such that $tw=\{{\left[start,end\right]}_{1},{\left[start,end\right]}_{2},\ldots {\left[start,end\right]}_{n}\}$ A duration of an event is defined using a value and a time unit, e.g., duration = 10 min.

If values for extent and granularity are omitted, *e* is relevant for any geographic area. If a value for time-window is omitted, *e* is relevant from the moment the geographic event was declared, and it has no expiration time. If a value for duration is omitted, *e* may have any duration.

**Example** **1.** *A user may define an a“air pollution” event as the occurrence of high concentrations of particulate matter (PM_10_) above $50\;\mu g/m^{3}$, and carbon monoxide (CO) above $10\;\;mg/m^{3}$, during a period longer than 10 min and within an area delimited by a polygon. Using Equation (1), we represent such geographic event as follows,*

$$\begin{array}{c} e=\{id:001,name:airpollution,phenomena:\left[PM_{10},CO\right],\\ conditions:PM_{10}>50\;\;\mu g/m^{3}\;\;AND\;\;CO>10\;\;mg/m^{3},\\ extent:POLYGON((30\;\;10,40\;\;40,20\;\;40,10\;\;20,30\;\;10)),\\ granularity:none,\mathrm{time}-\mathrm{window}:none,duration:10min\} \end{array}$$

**Detection rules**. A detection rule describes a pattern which must be matched by data streams during the detection processing step. We represent a detection rule using true statements (s), relational operators (•) (for instance, the logical operators AND and OR), temporal functions (f(t)) (for instance, *Duration()* or *Event Frequency()*), and spatial functions (f(l)) (for instance, *Intersect()*). A detection rule *r* is related to an event definition through the global event identifier; then, *r* is represented by the following general equation,

(2) $$r=\{id,\;\;\left(s_{1}\;\;•\;\;s_{2}\ldots \;•\;\;s_{i}\right),\;\;\left(f{\left(t\right)}_{1}\;\;•\;\;f{\left(t\right)}_{2}\cdots •\;\;f{\left(t\right)}_{j}\right),\;\;\left(f{\left(l\right)}_{1},\;\;•\;\;f{\left(l\right)}_{2}\cdots •f{\left(l\right)}_{k}\right)\}$$

**Example** **2.** *Using Equation (2), a detection rule for the geographic event defined in Example 1 is specified below. We omit some of the values for the sake of brevity.*

$$\begin{array}{c} r_{\left(air\;\;pollution\right)}=\{id,\;\;\left(PM_{10}>50\;\;\mu g/m^{3}\;\;AND\;\;CO>10\;\;mg/m^{3}\right),\\ (Duration\left(time1_{PM_{10}},time2_{PM_{10}}\right)\geq 10\;\;min\;\;AND\;\;Duration\left(time1_{CO},time2_{CO}\right)\geq 10\;min),\\ \left(Intersects\left(location_{PM_{10}},extent\right)\;\;AND\;\;Intersects\left(location_{CO},extent\right))\right\} \end{array}$$

**Notifications**. A notification describes the occurrence of a geographic event. A notification φ is represented by a set of values including: the global event identifier, an event name, the event time, and a payload containing a subset of values providing relevant metadata associated with the event (e.g., a list of the observations that generated the event). The payload of a notification is created during the *detection* step by fetching the list of observations that originate an event, such that

φ={id,name,time,payload};where

$$payload=[o_{1},o_{2},\ldots o_{n}]$$

**Example** **3.** *Assuming observations of PM_10_ and CO are collected every 5 min, a notification for our event example can be specified using a list of name-value pairs, such as*

$$\begin{array}{c} f=\{id:001,name:pollution,event\;time:2018-06-14\;12:30:00,payload:[\\ \{sensorID:100,phenomenon:{PM}_{10},measurment:52\;\;g/m^{3},\\ timestamp:2018-06-14\;12:15:00,location:POINT(10,30)\},\\ \{sensorID:200,phenomenon:CO,measurment:10\;\;mg/m^{3},\\ time:2018-06-14\;12:15:30,location:POINT(11,30)\},\\ \{sensorID:100,phenomenon:{PM}_{10},measurment:55\;\;g/m^{3},\\ timestamp:2018-06-14\;12:20:00,location:POINT(10,30)\},\\ \{sensorID:200,phenomenon:CO,measurment:12\;\;mg/m^{3},\\ time:2018-06-14\;12:20:30,location:POINT(11,30)\},\\ \{sensorID:100,phenomenon:{PM}_{10},measurment:53\;\;g/m^{3},\\ timestamp:2018-06-14\;12:25:00,location:POINT(10,30)\},\\ \{sensorID:200,phenomenon:CO,measurment:12\;\;mg/m^{3},\\ time:2018-06-14\;12:25:30,location:POINT(11,30)\}\;\;]\} \end{array}$$

## 4. RASCA: Reference Architecture for Smart City Applications

RASCA proposes a generic architecture as a framework for the implementation of the functionality required by event-driven applications. RASCA aims to comply with the following requirements:

1. Interoperability for consuming data from a variety of vendors and sensors, including in-situ, mobile and human sensors;
2. Reliable event processing capabilities for the detection of geographic events;
3. Real-time processing capabilities, especially to reduce delays in data transfer and processing to ensure a good quality of service; and
4. Scalability to cope with processing power required by applications deployed at a city scale, especially regarding the scenario where applications have to manage data from thousands of sensors and provide information to thousands of users.

**Figure 3:**
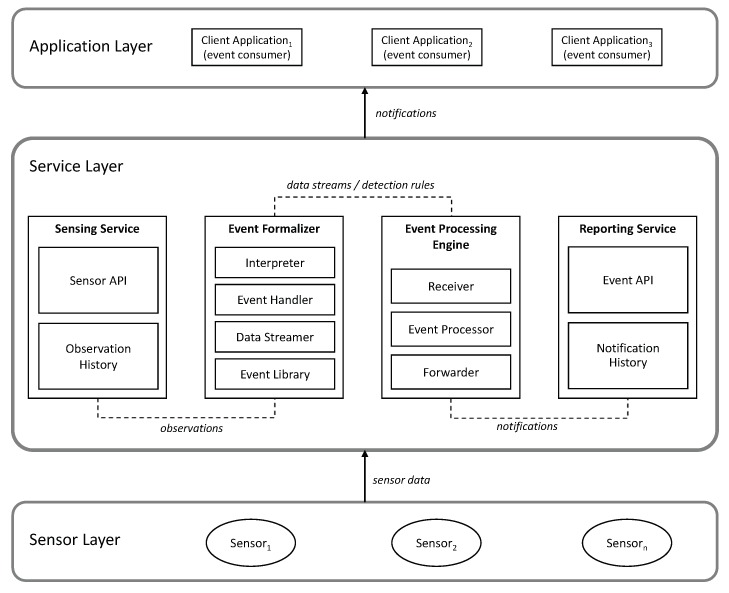
Layers and components of RASCA.

In the following sections, we describe the layers and component of RASCA, which are depicted in Figure 3.

### 4.1. Layered Architecture

RASCA consists of a sensor layer, a service layer, and an application layer. The *sensor layer* is responsible for collecting observations of dynamic geographic phenomena. This layer consists of in-situ, mobile, and human sensors. The *service layer* is responsible for realizing the processing steps of the event-driven processing workflow presented in Section 3.3. The components in this layer provide functionality to integrate data from sensors; to declare, formalize and detect geographic events; and to create notifications. The *application layer* is responsible for implementing client applications, which use notifications. Client spplications use notifications independently of one another, which means that event notifications can be used and reused by multiple clients.

The service layer serves as middleware between sensing devices and applications on the client side. This layer achieves data interoperability through the use of APIs; one API integrates sensors from multiple vendors, while another API pushes notifications to client applications. Event detection and real-time processing are achieved by implementing a service with CEP capabilities. Scalability is achieved through the modularity of the services. Because each service is independent, the components of this layer can be deployed as a decentralized system in a cloud computing environment. The expectation is that integration with cloud computing will guarantee scalability of the event-driven applications. The following section describes in detail each of the services and components inside the service layer.

### 4.2. Services and Components

The service layer (Figure 3) provides four independent services: sensing service, event formalizer, event processing engine, and reporting service. Each of these services encapsulates functionality required by the event-driven processing workflow.

The *sensing service* integrates and manages data from heterogeneous sensors. The *Sensor API* provides a standard interface for registering sensors and retrieving observations in real-time. This component also provides functionality for the selection of sensor based on spatial and temporal filters. The *observation history* provides access to historical records of sensors and observations registered in this service.

The *event formalizer* combines the functionality required by the event specification and formalization steps. The event formalizer provides functionality for the following tasks:

- Declaration and formalization of event definitions and detection rules *(interpreter)*;
- Managing the declaration and instantiation of events into the service *(event handler)*;
- Managing the flow of observations from the sensing service to the event processing engine *(data streamer)*; and
- Storing and indexing event definitions to be reused by different users *(event library)*.

The *Event Processing Engine* (EPE) detects geographic events over data streams. The EPE consist of three components: a receiver, an event processor, and a forwarder. The *receiver* provides a network interface for managing incoming data streams, and it establishes communication between the event formalizer and the EPE. The *event processor* applies filters and pattern matching on data retrieved from the sensors, in the forms of spatiotemporal functions and algorithms to detect geographic events. The *forwarder* creates and manage notifications using reports from the *event processor*.

The *reporting service* provides a subscribe–push service to manage notifications between the service layer and client applications in the application layer. In this service, an *Event API* provides a standard interface for subscribing to notifications of specific geographic events. The *notification history* includes functionality for archiving and retrieving historical notifications; this functionality is essential for client applications that require access to historical records of geographic events.

## 5. Geographic Event Detection System

The Geographic Event Detection System (GEDSys) is a system that complies with RASCA. GEDSys implements the service layer upon the SmartSantander sensor network (the sensor layer) and reports notifications to a client application entitled GeoSmart App (the application layer). Specifically, GEDSys implements the functionality required to:

- formalize geographic event definitions related to environmental phenomena (e.g., temperature);
- detect geographic events over data streams using the SmartSantander sensor network; and
- create notifications to report the occurrence of geographic events in real-time.

**Figure 4:**
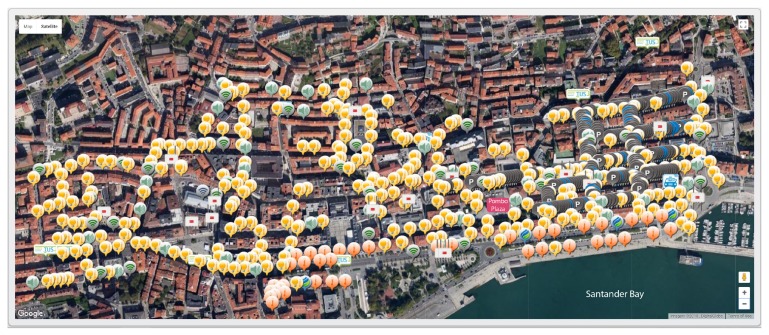
Screenshot showing some of the sensors (light, noise, temperature, and parking) deployed in the city center of Santander, Spain [86].

We use the case of Santander as a scenario for a real-world implementation. The SmartSantander sensor network is located in the city of Santander, Spain. The network is equipped with in-situ and mobile sensors that collect observations for environmental monitoring, traffic, parking, and irrigation. Figure 4 shows a screenshot of the location and distribution of some of the sensors. Light sensors (yellow icon) and noise sensors (green speaker) are scattered over the area; temperature sensors (reddish icon) are deployed along the harbor; and parking sensors (dark-grey and blue icons) concentrate to the east of Pombo Plaza. The following sections describe the development and implementation of GEDSys.

### 5.1. System Components

**Figure 5:**
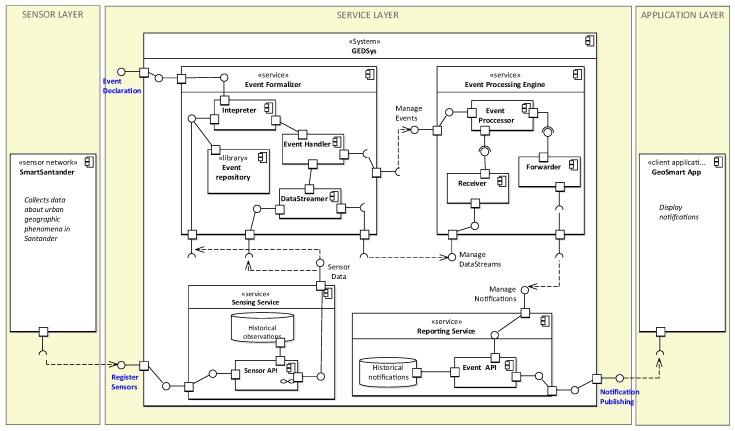
Component diagram of the Geographic Event Detection System (GEDSys).

The diagram in Figure 5 presents the components of GEDSys and its relations with SmartSantander and GeoSmart App. SmartSantader collects observations of urban geographic phenomena in the city of Santander, and the GeoSmart App displays notifications to end-users.

GEDsys exposes three interfaces for the exchange of information: register sensor data, event declaration, and notification publishing. Sensors are registered to the system using the *register sensors* interface. The *event declaration* interface allows declaring which geographic events will be detected by the system. Finally, the *notification publishing* interface allows client applications to subscribe to geographic events of interest and receive notification.

Several interfaces connect components inside GEDSys. The sensing and event formalizer services interact through the *sensor data* interface. This interface provides access to observations and metadata of sensors registered into the service. The Interpreter uses this interface to determine the availability of the data required for a geographic event. The data streamer uses the sensor data interface to request observations that meet the attributive, spatial, and temporal properties required for event definitions.

The event formalizer and EPE interact through two interfaces. The *manage events* interface gives the event handler access to configuration parameters of event processor. The event handler controls the instantiation of resources to detect events, i.e., valid data formats for the receiver and forwarder components, and detection rules in the event processor. *Manage datastreams* creates communication channels to push data streams into the EPE during runtime.

The *manage notifications* interface connects the EPE and the reporting service. Through this interface, the forwarder registers notifications into the Event API. Registered notifications are stored into the historical notifications repository, and made immediately available to GeoSmart App client through the notification publishing interface.

Components inside the services provided by GEDSys behave as follows. The register sensor interface registers sensors to the Sensor API and feeds observations into the system. When a geographic event is declared, the interpreter creates an event definition, which optionally is stored in the event repository for reuse. Then, the event handler deploys configuration parameters and detection rules into the event processor. The data streamer converts event definitions into data calls; it requests observations from the Sensor API and pushes them into the eeceiver.

The event processor executes detection rules and performs event detection. When events are detected, they are reported to the forwarder to create notifications. Notifications are registered into the Event API, which maintains a record of client applications that are subscribed to specific geographic events. Finally, the Event API pushes notifications to the GeoSmart App, where they are displayed.

### 5.2. Implementation

We built a prototype of GEDsys by integrating several technologies. For the Sensor API service, we used the FROST Server. This server implements the SensorThings API standard and defines a data model with eight entities: *things, sensors, locations, observations, sensors, observed properties, features of interest*, and *historical locations*. The SensorThings API also provides built-in spatial and temporal filters and query functions to retrieve data [87]. Sensor data are registered and retrieved from the FROST Server using HTTP requests.

The event formalizer component was implemented as several Python modules [88]. These modules automate the processes of instantiating an event in the system; they take event declarations as input; they validate event definitions by determining if the data required by the event definition is available in the Sensor API; and they automate the deployment of configuration parameters in the EPE. The event formalizer also controls the streaming of observations between the Sensor API and the EPE. Moreover, it implements a set of clean-up actions to free system resources when an event is deleted from GEDsys.

The EPE was implemented using the WSO2 Data Analytics Server version 3.1 [89]. This server implements the Siddhi CEP engine, a web-based complex event processing engine for stream processing, and it already implements the rest of the components of the EPE. The EPE uses the SiddhiQL language for specifying detection rules. SiddhiQL is a SQL-like language that provides a familiar syntax for writing queries [63].

The GeoSmart App was implemented using an HTTP client written as a Python module. In this first version of GEDSys, we did not implement the reporting service. Instead, we used a REST interface provided by WSO2DAS to push notifications directly to the GeoSmart App.

GEDsys implemented security features between the following components. Between the sensing service and the event formalizer, we implemented basic access authentication over HTTP, using user name and password; between the event formalizer and the EPE, we implemented SSH over TCP/P using public keys; and between the EPE and the reporting service, we used data encryption using HTTPS.

### 5.3. Instantiation of Components

**Figure 6:**
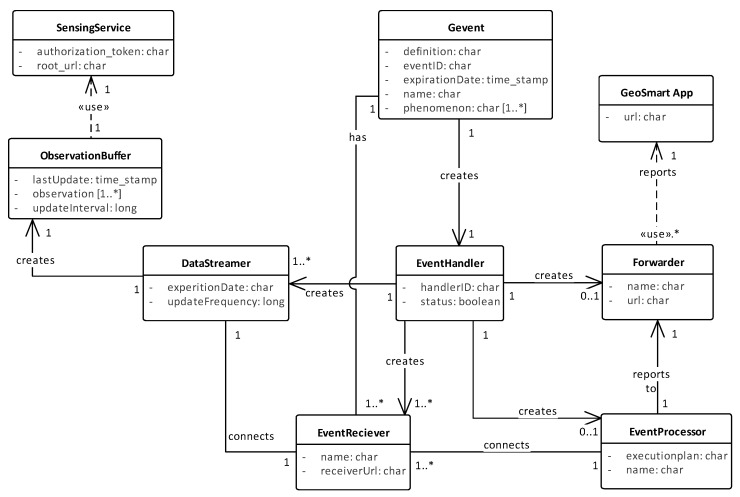
Entity model of the instantiation of components in the GEDsys prototype.

The entity model in Figure 6 depicts the instantiation of components in our implementation of GEDSys. This entity model describes how GEDSys behaves when implementing the steps of the event-driven processing workflow. Before any processing can be started, sensors and client applications should be registered into the system.

In the entity model, the *Gevent* entity encapsulates the declaration of a geographic event. The declaration of a geographic event into the system triggers the instantiation of the *event handler*, which instantiates a *data streamer* for each phenomenon declared in the event definition.

The *observation buffer* requests observations from the *sensing service* using batch requests. The *event handler* instantiates the *event receiver*, *event processor* and *forwarder* entities. The declaration of a geographic event does not instantiate the *sensing service* or the *GeoSmart App* entities. Instead, they are used by the processing workflow to access observations and to report notifications, respectively.

### 5.4. Data and Deployment

For our GEDSys prototype, we used data from the SmartSantander sensor network. We harvested three years of observations (2014–2017) from their map portal. We loaded one month of data (July 2016) into the sensing service, which contains 2337 sensors and ∼5.5 million observations for luminosity, temperature, noise, carbon monoxide, particulate matter, and 14 other geographic phenomena.

We deployed our GEDsys prototype using two machines. The sensor service and the EPE were deployed in a machine with eight cores and 8 GB of RAM running Ubuntu Server, while the event formalizer and the GeoSmart App were deployed in a machine with two processing cores and 8 GB running Windows. This set-up allowed testing the system in a distributed processing environment.

## 6. Performance Testing and Results

Our GEDSys prototype was tested to determine how it responds to the detection of geographic events. During the testing, we focused on the following questions:

1. Can the system detect and report the occurrence of geographic events in the SmartSantander?
2. How does the system respond to a high throughput of data streams when detecting simple geographic events?
3. How does the system respond to the declaration and detection of many geographic events?

We tested two scenarios to answer the questions. The first scenario addressed Questions 1 and 2, and the second scenario addressed Question 3. In the following sections, we present and discuss the results.

### 6.1. Scenario 1. Detection and Data Throughput

For this scenario, we defined a simple geographic event (*T*) for temperatures above 0 °C and an area around the center of Santander city that included 626 temperature sensors, and a time window between 10:00 and 11:00 on 24 of November 2016. An arbitrary value of zero degrees was chosen to maximize the number of data streams that match the detection rules; with this value, all data streams should match the detection rules.

The “temperature” event (T) was specified as shown below. The values for the extent property are expressed in longitude and latitude.

$$\begin{array}{c} e=\{name:temperature,phenomena:[Temperature],condition:Temperature>0\;{}^{{}^{\circ}}C,\\ extent:POLYGON((-3.8469736\;\;43.4414848,-3.84669736\;\;43.4863448,\\ -3.76632358\;\;43.4863448,-3.7663236\;\;43.4414848,-3.8469736\;\;43.4414848)),\\ \mathrm{time}-\mathrm{window}:2016--11--24T10:00:00Z/2016--11--24T11:00:00Z\} \end{array}$$

We determined the successful detection of (T) by monitoring the number of notifications displayed by the GeoSmart App. We accounted for the response of GEDsys to a high data throughput by controlling the number of data streams processed through the system. We used the 626 sensors to generate the same amount of data streams, each containing a single observation. Then, we used CPU threading in the formalizer service to gradually increase the number of concurrent data streams sent to EPE.

**Figure 7:**
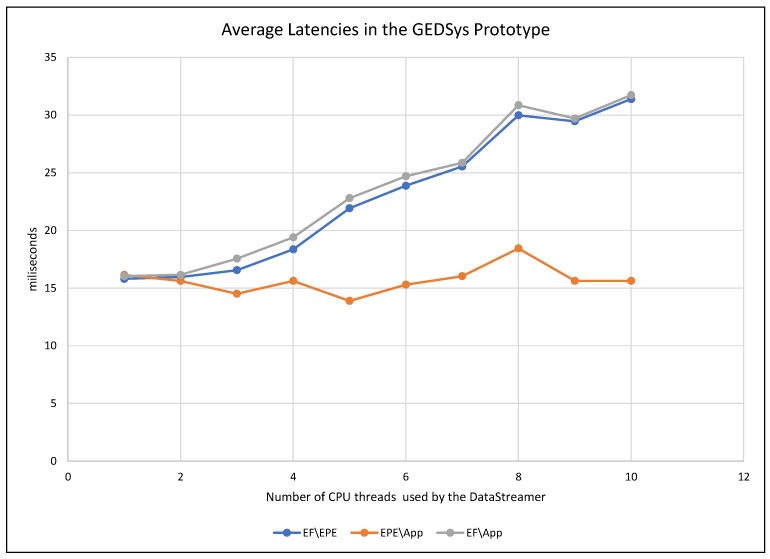
Average latencies in GEDSys caused by the increment of the number of concurrent data streams.

**Figure 8:**
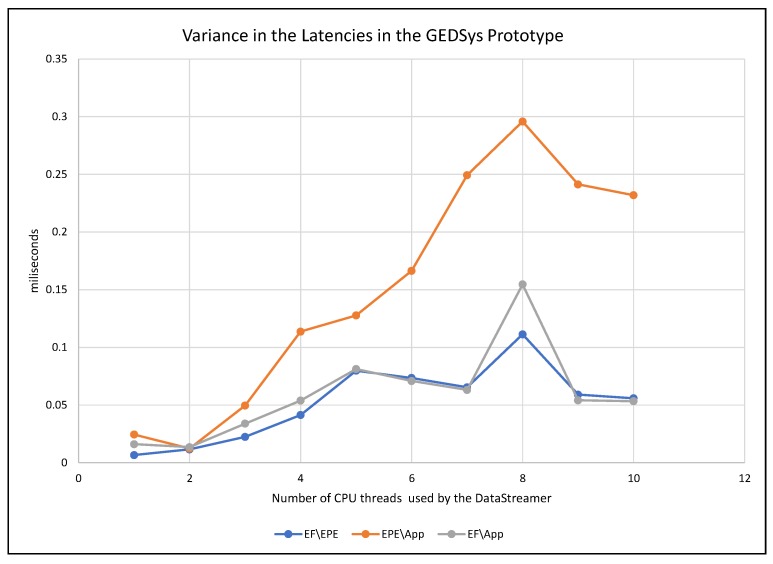
Variance in the latencies in GEDSys caused by the increment of the number of concurrent data streams.

To monitor the response of the system to the increment of data streams, we measured the latency in three points of GEDSsys: (1) between the event formalizer and the EPE services (EF\EPE); (2) from the EPE to the GeoSmart App (EPE\App); and (3) the overall latency, i.e., the time between the dispatch of data streams by the event formalizer and the arrival of notifications at the GeoSmart App (EF\App). Figure 7 shows the average latency through the components of GEDSys during the detection of (T), and Figure 8 presents the variance of the latencies.

GEDsys showed an increment in latency between the event formalizer and the EPE when increasing the number of concurrent data streams. This increment is caused by delays that resulted as a consequence of using a single port to receive data streams in the EPE. As a consequence of the increment of the (EF\EPE) latencies, the overall latency increased proportionally. The latency in the EPE did not change significantly; this confirms the claims that WSO2DAS is a high-performance CEP engine.

The variances in latencies were generally low, with values between 0.007 and 0.3 ms (see Figure 8). The most significant variances in latency occurred between the EPE and the GeoSmart App (EPE\App). This behavior is explained by the fact that during this test the GeoSmart App was using a single thread to receive notifications, which caused notifications to queue before the GeoSmart App could display them. The latencies on the communication network also played a role in the delays shown here.

### 6.2. Scenario 2. Multiple Geographic Events

The second scenario aimed to determine how our prototype handles the instantiation and detection of multiple geographic events. For this scenario, we used multiple instances of event *T* and measured the average latencies in the system between the same points described in the previous scenario (Section 6.1). We also measured the data buffering time, i.e., the time that it takes to retrieve observations for all the events handled by the system from the Sensor API, and the total time required to configure the EPE for all geographic events, also called deployment time.

**Figure 9:**
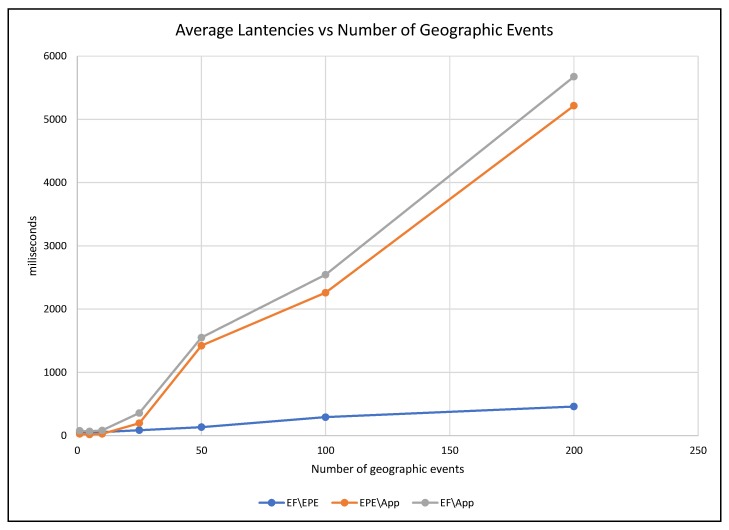
Average latencies caused by instantiating multiple geographic events in GEDSys.

The average latency between the components of GEDsys increased as a result of increasing the number of geographic events (Figure 9). The latency between the event formalizer and the EPE increased from 49 ms (with one event) to 459 ms (with 200 events). The latency between the EPE and the GeoSmart App increased dramatically, up to 5217 ms when handling 200 events. A contributing factor to such increment may be the delays generated by GeoSmart App when handling many concurrent notifications. However, delays in this component can be reduced by using more efficient thread management or replication.

**Figure 10:**
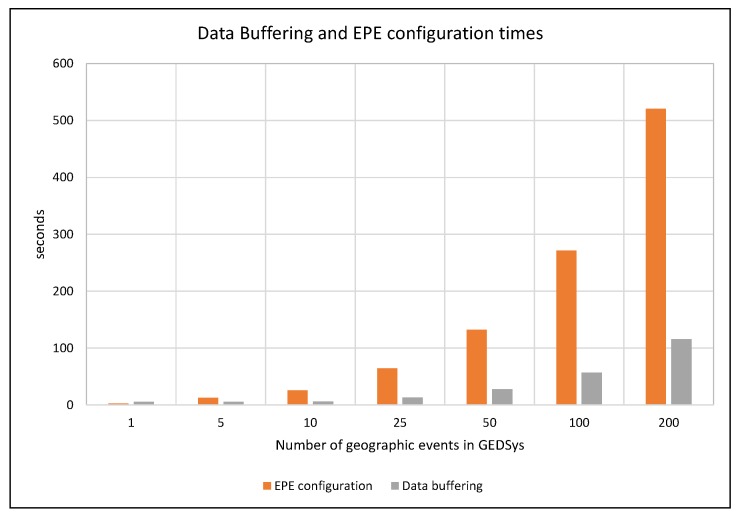
The effect of the number of geographic events in GEDSys on data buffering and EPE configuration times.

Data buffering and EPE configuration times increased with the number of geographic events instantiated in GEDSys (Figure 10). The time required for configuring the EPE showed an exponential growth; it doubled when increasing the number of events handled by the system. Such behavior was expected because these components implemented using synchronous communication, i.e., the event formalizer transfers configuration files to the EPE one at a time for each geographic event declared in the system.

Data buffering showed a similar trend with a less pronounced growth. Data buffering was executed asynchronously; this reduced the time required to fetch observations for all events in the system. This also means that hardware resources condition the performance of data buffering (e.g., number of cores in the CPU), and the response time of the sensing service.

## 7. Discussion

In this section, we compare our architecture with other reference framework. We compare our implementation of GEDSys with current event processing systems that share similar functionality, and then we discuss the result of our performance tests. Finally, we explain and address some of the limitations of our system prototype and the test scenarios.

### 7.1. Implementation and Performance Testing

RASCA describes an architecture that complies with most of the requirements of platforms for smart cities. When compared with architectural characteristics in Table 1, RASCA complies with all but two of the requirements: batch processing and historical analysis. These two requirements are interrelated, i.e., to implement historical analysis, batch processing capabilities is indispensable. However, this is expected because the objective of event-driven applications relies almost exclusively on real-time processing and analysis. Nevertheless, the reporting service would benefit from implementing batch processing and historical analysis, for instance, to provide information about the spatial and temporal pattern of the occurrence of geographic events.

When compared with similar CEP systems, GEDSys adds spatial functionality to traditional CEP systems and enables the detection of geographic events. Apache Flink and LIDAS are relatively new stream processing systems that also implement CEP. Apache Flink is a system that offers both stream processing and batch processing and features good fault tolerance mechanisms [68]. LIDAS (Lightweight Intelligent Data Analytics System) implements the LIDA-E CEP engine, and was developed to apply CEP to the monitoring of distributed systems [70]. However, none of these systems currently offer the spatial matching functionality required for the detection of geographic events. To the best of our knowledge, only the work in [32] proposes a system with a focus on geographic events. However, their system is able to implement only geofencing as spatial functionality. Additionally, the spatial functionality provided by GEDSys can be further extended, for instance by adding spatial operators that aggregate geographic events based on their spatial proximity.

GEDsys achieved interoperability between the sensor and service layers by adopting open standards. Further development is required to implement interoperability between the service and application layers through the Event API. To achieve this, we will rely on industry standards to implement communications protocols and formatting of the notifications, such as HTTP and JSON.

Thanks to the modularity of its architecture, GEDSys was deployed in a decentralized environment. This characteristic provides scalability to event-driven applications, but it may create possible points of failure and reduce fault tolerance, especially during communication between components. However, the use of global IDs for identifying event definitions, detection rules, and notifications combined with precise logging of the state of the system does offer basic mechanisms for fault tolerance. Additionally, GEDSys could implement checkpoints as a mechanism to recover the state of the system after a failure, thus increasing its fault tolerance.

The performance tests demonstrated that GEDSys is capable of detecting geographic events in the SmartSantander network, and the system can handle a high throughput of concurrent data streams. However, two factors influenced the results presented in Section 6.1 and Section 6.2: the workload in the system components and the capacity of the communication network [70]. The effect of the workload on the performance was evident during EPE configuration and data buffering. The times for completing such routines increased with the number of geographic events instantiated in the system. The network capacity influenced the results shown in Figure 9, and it was responsible for the slight increment in latencies between the event formalizer and EPE. GEDSys showed more significant latencies, longer data buffering times, and longer EPE configuration times when handling multiple geographic events. In contrast, the event formalizer showed a small increment in latency. Communication latencies during the deployment of configuration files are explained by the use of synchronous communication, between the event formalizer and the EPE.

### 7.2. Limitations

Through the implementation and testing of GEDSys, we demonstrated its capabilities to detect simple geographic events. However, our current implementation does not provide enough functionality to detect composite events. There are two reasons for this. First, the definition composite events required a very expressive mechanism to represent advance spatial and temporal relations between simple events, and consequently expressive spatial and temporal operators and advanced functions to construct detection rules. Second, the technical challenges of implementing the logic required detecting composite events. These are not new to experts in the field of complex event processing, but neither is trivial to address, and the existing solution present performance concerns (e.g., [62,90]). Nevertheless, the works by Cugola et al. [61] and Ghezzi et al. [91] provide fundamental knowledge to design solutions to these limitations.

Because our implementation of GEDsys relies on an existing event processing engine, we did not test more advanced scenarios. We could also not thoroughly test the scalability of our GEDSys, because our implementation platform did not provide the required computational power for such cases. Migrating the current implementation to a cloud computing environment should give enough flexibility to evaluate city scale scenarios.

GEDSys provides security only among its internal components and at a basic level. However, the implementation of advanced security features should not pose big challenges because the system was implemented upon technologies known for their security features. Security between GEDSys and exterior components, i.e., sensor networks and client applications, can be implemented using token-based authentication and AppKey-based encryption.

## 8. Conclusions and Future Work

This work provides a generic approach for designing and developing information services in smart cities. The approach provides developers with a generic processing workflow for the detection of geographic events, and a reference architecture to develop event-driven applications.

Despite the current limitations of our implementation of GEDSys, the prototype serves as a proof of concept for RASCA. RASCA and GEDSys successfully integrate IoT technology, CEP, and spatial analysis for the implementation of smart city applications that rely on the detection of geographic events. Performance tests of the GEDSys prototype showed its capabilities to detect geographic events using sensor networks. The performance tests also demonstrated the potential of the GEDsys to support scalable and reliable applications.

In the future, we aim to extend GEDSys and evaluate its performance for the detection of composite geographic events, to identify strategies to reduce latencies in its components, and to implement advanced fault tolerance strategies. We plan to minimize latencies in the sensing service and Sensor API by implementing better memory management and more accurate database indexing. Latencies in the configuration of the EPE can be reduced by performing asynchronous communication with the event formalizer, and the use of replication on the EPE could provide robustness to GEDSys. Fault tolerance will be implemented in the system after identifying suitable strategies through literature review.

We also plan to extend the testing scenarios to assess the performance of the system at a smart city scale. For this reason, we are currently migrating our implementation to a cloud computing environment in which we can assess how GEDSys responds when scaling up the number of simulated events, and assess its performance in a city scale scenario.

We also plan to use RASCA and GEDSys to develop other application cases. We are currently working on the design of two applications. The first, a recommender system for runners, that detects high concentrations of air pollution to suggest training routes. The second a smart transport management system that uses the detection of rain and the number of passengers in bus stops to manage the frequency of buses.

## Figures and Tables

**Table 1:** Architectural characteristics of smart city platforms for the exploitation of data from sensors.

Architecture	Service Oriented	Decentralized Computing	Scalability	Security and Privacy	Batch Processing	Real-Time Processing	Historical Analysis	Real-Time Analysis
Suciu, G. et al. [45]	✓	✓	✓		✓	✓		
Shaikh, T. et al. [46]	✓	✓		✓				
Clement, S. J. et al. [47]	✓	✓					✓	✓
Santana, E. at al. [43]	✓	✓	✓	✓	✓	✓	✓	✓
Shahat Osman, A. [44]	✓	✓	✓	✓	✓	✓	✓	✓

**Table 2:** Functionality of open source, general-purpose CEP engines.

CEP Engine	Data Model	Temporal Matching	Spatial Matching	Atrribute Matching	Performance (Throughput)
SASE [64]	Heterogeneous	✓		✓	n.p.
T-Rex [62]	Heterogeneous	✓	partial	✓	100 K/s to 1 M/s
Esper [65,66]	Homogeneous	✓		✓	100 K/s
Kinetic Rule Engine (KRL) [60]	Heterogeneous	✓		✓	n.p.
Siddhi CEP [63]	Heterogeneous	✓	partial	✓	800 K/s to 1.2 M/s
Caguya [67]	Homogeneous	partial		✓	n.p.
Apache Flink [68,69]	Heterogeneous	✓		✓	1.5 M/s
LIDA-E [70]	Heterogeneous	✓		✓	20 K/s

**Table 3:** Roles and motivations of users of event-driven applications.

Role	Motivation
City Administrator	city monitoring, emergency response and planning
Businessman	business opportunities and strategies
Citizen	individual’s need and desires

## Abbreviations

The following abbreviations are used in this manuscript:

IoT	Internet of Things
RASCA	Reference Architecture for Smart City Applications
SWE	Sensor Web Enablement
SOS	Sensor Observation Service
API	Application Programming Interface
CEP	Complex Event Processing
n.p.	Not provided
PM_10_	Particulate matter
CO	Carbon monoxide
EF	Event Formalizer
EPE	Event Processing Engine
GEDSys	Geographic Event Detection System
FROST Server	FRaunhofer Opensource SensorThings-Server
WSO2DAS	WSO2 Data Analytics Server
HTTP	Hypertext Transfer Protocol
HTTPS	HyperText Transfer Protocol Secure
TCP/IP	Transmission Control Protocol/Internet Protocol
SSH	Secure Shell
LIDAS	Lightweight Intelligent Data Analytics System
